# Diseases of Gastropoda

**DOI:** 10.3389/fimmu.2021.802920

**Published:** 2022-01-13

**Authors:** Michelle F. O’Brien, Sarah Pellett

**Affiliations:** ^1^ Wildfowl & Wetlands Trust, Gloucestershire, United Kingdom; ^2^ Animates Veterinary Clinic, Thurlby, United Kingdom

**Keywords:** gastropod disease, mollusc, virus, bacteria, fungi

## Abstract

Gastropods (class Gastropoda) form the largest of the classes in the phylum Mollusca and inhabit terrestrial, fresh water and marine environments. A large number of these species are of major conservation importance and are an essential component of ecosystems. Gastropods may be deemed as pests, having a negative impact in horticulture and agriculture, whereas others may be used as a food source for human consumption and therefore are beneficial. Gastropods are susceptible to primary diseases and also act as intermediate hosts for diseases which affect other animals, including humans. The diseases described include two that are notifiable to the World Organisation for Animal Health (OIE): *Xenohaliotis californiensis* and Abalone viral ganglioneuritis caused by Haliotid herpesvirus-1 (HaHV-1). Research into the diseases of gastropods has often focused on those species that act as intermediate disease hosts, those that are used in research or those cultured for food. In this paper we review the viral, bacterial, fungal, parasitic and miscellaneous conditions that have been reported in gastropods and mention some of the factors that appear to predispose them to disease. The pathogenicity of a number of these conditions has not been fully ascertained and more research is needed into specifying both the etiological agent and significance in some of the diseases reported.

## Introduction

Gastropods (class Gastropoda, phylum Mollusca) are comprised of more than 80,000 species and are differentiated from other classes of mollusca by the presence of a torsed body. They are separated into three subclasses: Prosobranchia, Opistobranchia, and Pulmonata. They are found in terrestrial, freshwater and marine environments. All gastropods possess a ventrally flattened foot that provides locomotion (1). Over 2000 species are reported in the International Union for Conservation of Nature (IUCN) Red List as critically endangered, endangered or vulnerable, with 14 species listed as extinct in the wild. Over 1500 other species cannot be classified due to a deficiency in data (2).

Gastropods can be used in a number of different types of research including animal and human parasites and neurobiological research (1). With such a diverse class of animals, environmental requirements differ greatly and can often be species specific. Gastopods can become predisposed to diseases due to living in adverse environmental conditions and therefore individuals dealing with them in captivity should be aware of the individual temperature, humidity, nutrition and aquarium/terrarium design requirements for their particular species. Investigations into infectious diseases of gastropods have often centred on those species cultivated as food or those that act as vectors for zoonotic diseases. A sequelae to gastropods becoming more prevalent in the pet trade and zoological collections is likely to be advancement into diagnosis and treatment of their diseases.

## Viruses

A number of viral infections have been reported in gastropods although more research is needed in many cases to identify the specific virus. Abalone viral ganglioneuritis caused by Haliotid herpesvirus-1 (HaHV-1), has been reported in farmed and free-living abalone *Haliotis rubra*, *Haliotis laevigata* and hybrid *H. laevigata* x *H. rubra* in Australia and cultured *Haliotis diversicolor supertexta* cohabiting with *Haliotis cracherodii*, in Taiwan. This disease is reportable to World Organisation for Animal Health (OIE) and the US Department of Agriculture (USDA) (3). High mortality rates (up to 90%) have been reported and death often occurs within 1-2 days (3). Histological signs indicative of intranuclear inclusion bodies, may be seen in the neurons. Confirmation is by conventional and real-time PCR (4, 5).

Following a mass mortality event in *Theba pisana* (an intermediate host in human and veterinary medicine), transmission electron microscopy (TEM) confirmed nuclear inclusions where unenveloped, roundish virus-like particles were observed (6) although the causal virus was not identified.

Viruses from a number of families have been identified in snails, abalone and whelks including: Bacilladnaviridae (The International Committee on Taxonomy of Viruses (7), Circoviridae, Reoviridae, Picornaviridae, Caliciviridae, Paramyxoviridae and Rhabdoviridae (8).

## Bacteria

It can be complex to determine the presence of bacteria in molluscs as pathogenic as a number of species harbour a large number of commensal bacteria (9). Commensal bacteria have also been shown to be a likely source of Tetrodotoxin in *Nassarius semiplicatus* (10). Diseases caused by bacteria will often present differently depending on the life stage affected, with larval stages often showing high mortality whereas fewer diseases of adults have been reported (9).

*Xenohaliotis californiensis*, a Rickettsial- like prokaryote (RLP), causes withering syndrome in abalone. This disease is notifiable to the OIE (11). The organism invades the digestive gland and the animal exhibits a loss of condition and atrophy of the foot muscle. In laboratory studies, time from infection to signs of disease was 245 days and *Haliotis cracherodii* was more severely affected than *Haliotis rufescens*. Transmission is direct between individuals (12). *Haliotis corrugata* and *Haliotis fulgens* (13) seem to be more resistant than other abalone species (14, 15). Oxytetracycline injections have been shown to halt progression of the disease in treated animals (16). Rickettsial infections in *Haliotis diversicolor supertexta* (17), caused similar symptoms, but symptoms and mortality only occurred at water temperatures of 30°C. (18) also showed that at least 2 genetic variants show a different host specificity. *Vibrio parahaemolyticus* has also been isolated from *H. diversicolor supertexta* in Taiwan showing signs of withering syndrome (19, 20).

*Vibrio harveyi* has caused up to 80% mortality of wild and cultured *Haliotis tuberculata* on the coast of France (21). Travers et al. (22) showed that infection was linked to ripe or just spawned individuals in water temperatures above 18°C. Juveniles did not develop disease (23), suggested that this disease occurrence may be linked to global warming.

Potentially zoonotic bacteria have been isolated from Giant African land snails, although they do not appear to cause disease in these animals (24). *P. putida* and *C. indologenes* have been associated with infections, such as bacteraemia, in hospitalised patients (25, 26).

*Biomphalaria* sp. are an intermediate host for *Schistosoma* sp. therefore they have been the focus of substantial research into molluscicidal bacteria (27).

Mycobacteria have shown pathogenic activity (28) and experimental transmission has been shown to 6 species of fresh water snails. See **Table 1** for further details of bacterial diseases.

**Table 1:** Bacterial diseases of gastropods.

Species	Class of pathogen	Name of pathogen	Signs and symptoms	References
*Haliotis* spp.	Rickettsiales- like prokaryote (RLP)	*Xenohaliotis californiensis*	Withering syndrome	(13, 16, 17, 29–32)
*Haliotis* spp.	Stippled RLP	Unclassified	Non pathogenic	(3)
*Haliotis tuberculata*	Gram negative bacterium	*Vibrio harveyi*	80% mortality	(21)
				(33)
*Haliotis diversicolor supertexta*	Gram negative bacterium	*Vibrio parahaemolyticus* and *Vibrio alginolyticus*	Post larval and adult mortality	(19, 34–38)
*Haliotis rufescens*	Gram negative bacterium	*Vibrio alginolyticus*	Larvae and post larval mortality	(39)
*Haliotis tuberculata*	Gram negative bacterium	*Vibrio harveyi*	Mortality	(40)
*Haliotis asinina*	Gram negative bacterium	*Vibrio vulnificus Vibrio alginolyticus*	Mortality	(41)
*Haliotis rubra, Haliotis laevigata*	Gram negative bacterium	*Vibrio splendidus*	Mortality	(42)
		*Vibrio harveyi*		
*Haliotis tuberculata*	Gram negative bacterium	*Vibrio splendidus*	Mortality	(43)
*Haliotis tuberculata*	Gram negative bacterium	*Vibrio tubiashii*	Mortality	(44)
*Haliotis discus hannai*	Gram negative bacterium	Unclassified	septicopyaemia	(45)
*Haliotis midae*	Gram negative bacterium	*Vibrio* sp.	Mortality	(46)
*Haliotis asinina*	Gram negative bacterium	*Pasteurella* sp.	Mortality	(41)
*Haliotis rubra, Haliotis laevigata*		Flavobacterium-like bacteria	Epithelial disease	(42)
*Haliotis diversicolor supertexta*	Gram negative bacterium	*Shewanella alga*	Postlarval mass mortality	(47)
*Haliotis diversicolor supertexta*	Gram negative bacterium	*Klebsiella oxytoca*	Acute mortality larvae and postlarval juveniles	(48)
*Haliotis* sp.	Gram positive bacterium	*Clostridium lituseberense*		(49, 50)
*Haliotis midae*	Gram positive bacterium	*Clostridium* sp.	Mortality	(46)
*Haliotis gigantea*	Gram negative bacterium	*Francisella halioticida*	Mortality and loss of adhesive strength	(51, 52)
*Achatina fulica*	Gram negative bacterium	*Aeromonas hydrophila*	Skin lesions, Cellulitis likely secondary to abrasion	(53, 54)

*Biomphalaria glabrata*	Gram positive bacterium	*Candidatus Paenibacillus glabratella*	Mass mortality and decreased egg hatching	(27)
*Biomphalaria glabrata*	Gram positive bacterium	*Bacillus thuringiensis kurtsaki*	Mortality and decreased egg hatching	(55)
*Biomphalaria glabrata*	Gram positive bacterium	*Brevibacillus laterosporus*	Pathogenic in juveniles (toxicity increased in younger larvae)	(56)
*Biomphalaria pfeifferi* and *Bulinus truncates*	Gram positive bacterium	*Bacillus brevis*	Pathogenic in laboratory setting	(57)
*Helisoma anceps*	Mycobacteria	Unclassified	Pathogenic	(28)
*Biomphalaria glabrata*	Mycobacteria	Unclassified	Tumour – no obvious pathogenicity	(58, 59)
*Bulinus jousseaumei*	Gram negative bacterium	Unclassified	Tumour – no obvious pathogenicity	(60)

## Fungi

Only a small number of fungal conditions have been reported in the literature affecting gastropods and only minimal information is available in some cases (61), reported fungal disease (potentially linked to shell boring invertebrates) causing lesions on the inside of the shell in *Haliotis iris, Haliotis australis* and *Haliotis virginea*. The shell length of affected animals was significantly smaller than those unaffected and fatalities occurred in captive animals. The fungus has only been provisionally suggested as *Deuteromycotina* (62).

A fungal disease in *Haliotis sieboldii*, in Japan, showed tubercle-like swelling on the mantle and melanized lesions on the peduncle. This fungus was designated *Atkinsiella awabi* sp. nov. (63). *Haliotis midae, Haliotis rufescens* and *Haliotis sieboldii*, in Japan also showed white nodules on the mantle and mortality due to *Halioticida noduliformans* gen. et sp. nov. by phylogenetic analysis (64).

(65) also reported lung nodules in *Pomacea canaliculata* likely caused by *Poterioochromonas* sp., a species of golden algae, although the pathogenicity of this finding was unclear.

Fungal disease has also been reported in *Haliotis sieboldii* including *Haliphthoros milfoldensis* (66), *Halocrusticida awabi* (63) and *Atkinsiella dubia* (67) The mycelium was always observed in the lesions of diseased abalone with flat or tubercle-like swelling (68).

## Parasites

### Ectoparasites

Mites can parasitise Giant African land snails and their pathogenicity varies with the species (69). High mite burdens can lead to debilitation of the snail.

### Endoparasites

#### Protozoa

*Pseudoklossia patellae*, a coccidian, has been detected in the epithelial cells of the intestine, kidney, and digestive cells of the limpet, *Patella vulgata*, prosobranchs and *Haliotis* spp. (70, 71). However, the intermediate hosts from gastropods are not known and no treatment has been described (70).

Ciliates were observed in tissues of two nudibranchs. A flagellate parasitising egg masses of doridacean nudibranchs and a parasite in the Thraustochytriaceae family of marine protists, producing Yellow-spot disease in *Tritonia diomedia*, a dendronotacean nudibranch (72). This family of marine protists is frequently included in the lower fungi. The amoebocytes of the gastropod become flattened and form a lamellated wall around the parasitic cells to form a necrotic thick-walled acellular capsule (72).

Invertebrates are often intermediate hosts for a variety of metazoan parasites with gastropods being one of the genera that *Licnophora* spp. infests. Opportunistic infections may arise and their importance in causing disease varies on environmental conditions and the host species (3).

*Partula turgida*, is especially notable as it succumbed to a microsporidian parasite (*Steinhausia* sp.), and is claimed to be the first extinction caused by an infectious disease (73). The colony had declined from 296 individuals over 21 months, and post-mortem examinations showed the microsporidian present, although absence from other species suggested that it might be specific: indeed, Cunningham and Daszak raised the possibility that the parasite might also have caused its own demise.

#### Annelida

Shells of abalone may be infested by Annelids, worms that burrow into the matrix of the host’s shell and form tunnels, compromising the host shell’s protective and supportive functions (3).

#### Nematoda and Trematoda

Gastropod-borne parasites may be of concern for human and animal health. *Cornu aspersum*, an edible gastropod of Mediterranean origin, is an intermediate host for several metastrongylid nematodes (74–77). Of veterinary significance, is the increasing number of cases of *Angiostrongylus vasorum*, the dog lungworm, seen in the UK because of a decline in preventative treatment in 2020 as a result of Covid-19 restrictions (78). Dogs become infected by ingesting slugs and snails which are the intermediate hosts.

Gastropods are also sole hosts of Rhabditida, Mermithida and Ascaridida nematodes (77). The opportunistic parasite of slugs, *Phasmarhabditis hermaphrodita* has been formulated and developed into a biocontrol agent against slugs and commercialised.

Aquatic gastropods contribute to the distribution of trematodes, e.g. Schistosoma, that risk human health. The infectivity of *Schistosoma mansoni* to *Biomphalaria glabrata* has been shown to vary depending on life stage of the snail (79) and temperature (80). *Brachylaima*, an avian trematode, transmitted by the gastropod *Monacha*, is also zoonotic (81).

Occasionally gastropods may serve as a final host for trematodes and these parasites can be seen in the kidney or the lumen of the digestive gland e.g. *Proctoeces buccini* was described in the nephridial lumen of the dog whelk (70, 82).

#### Turbellaria

Turbellariad (flatworm) infections have been described in the haemocoel of aquaria held gastropods (1) and in the dilated renal lumen and mantle cavity in free-living dog whelk in the North Sea (70).

#### Copepoda

Splanchnotrophidae are endoparasitic copepods and can affect nudibranchs by producing egg sacs under the external body wall, or which project through the host’s body wall (1). There is paucity in the literature describing lesions and mortality caused by these parasites. See **Table 2** for further details of bacterial diseases.

**Table 2:** Parasitic diseases of gastropods.

Species	Class of pathogen	Name of pathogen	Signs and symptoms	References
*Helix* spp.	Mite	*Riccardoella limacum*	Lung pathology	(83)
			Poor growth	
*Haliotis* spp.	Renal coccidian	*Margolisiella haliotis*	Often asymptomatic although epithelial cell hypertrophy is detected. Seen in individuals also affected with withering syndrome.	(1, 3, 84)
*Buccinum undatum*	Renal coccidian	*Merocystis kathae*	Often asymptomatic although epithelial cell hypertrophy is detected.	(1, 3, 84)
*Haliotis* spp.	Coccidian	*Pseudoklossia patellae*	Unknown	(70, 71)
*Patella vulgata*				
*Tritonia diomedia*	Marine protist	Thraustochytriaceae family	Yellow-spot disease	(72)
Juvenile *Haliotis* spp. < 90 days of age	Protozoan parasites	Labyrinthula protozoan parasites	Mortality	(85)
*Hermissenda* nudibranchs	Ciliate	*Licnophora* spp.	Parapodial mantle-gill complex	(86)
*Aplysia* sea slugs			Branchitis	
			Dermal ulcers	
*Haliotis iris*	Sporozoan parasites	*Haplosporidium* spp.	Mortality in juvenile culture stocks. In wild caught adults infection was present but no clinical disease.	(1, 87)
	Plasmodia			
Juvenile *Haliotis tuberculata*	Plasmodia	*Haplosporidium montforti*	Discolouration of foot.	(88, 89)
			Lack of adherence to surface. Linked to rise in water temperature.	
*Partula turgida*	Microsporidian	*Steinhausia* spp.	Mortality	(73)
*Haliotis* spp.	Annelids. Polychaeta	*Polydora* spp.	Damage to matrix	(3)
		*Boccardia* spp.		
*Haliotis rufescens*	Polychaeta	Sabellid polychaete	Malformed shell	(1, 90)
*Achatina* spp.	Nematode	*Angiostrongylus cantonensis*	L3 larva can be zoonotic, causing eosinophilic meningoencephalitis	(91–95)
Slugs and snails	Nematode	*Aelurostrongylus abstrusus*	Intermediate host for dog, cat and fox lungworms	(96)
		*Angiostrongylus vasorum*		
		*Crenosoma vulpis*		
*Milax sowerbyi*	Nematode	*Phasmarhabditis apuliae*	Facultative mollusc-parasites - survive long-term in decaying organic material (saprophytic phase)	(97–99)
*Milax gagates*				
Marine snails	Trematode	Digenetic trematodes	Giganticism	(70, 100)
			Colour changes	
			Behavioural changes	
			Shell changes	
			Intermediate host – zoonotic potential	
*Hermissenda crassicornis*	Flatworm	Turbellariad flatworm	Mortality	(1)

## Other

Shell lesions of unknown etiology, leading to reduced growth rate, have been reported in *Haliotis iris* (101).

Gas bubble disease was reported by (86) in *Aplysia* caused by exposure to seawater supersaturated with air. Air bubbles have also been identified in the body and cerata of captive *Hermissenda*. Death (probably caused by pressure necrosis of vital organs by the air bubbles) usually occurred (102).

Neoplasia is rarely described in invertebrates, but has been reported in *H.discus* (glioma of the pleuropedal nerve cord), *Ampullarius australis (*papilloma of the epidermis and adenoma of the digestive gland) and *Chiton tuberculatus* (papilloma of the gastrointestinal tract) (103).

Parry and Pipe (104) found that exposure to three stressors (copper, temperature, bacteria) could alter certain aspects of molluscan immune function and produce complex results.

## Discussion

Anthropogenic activities that pollute the environment can affect molluscan physical parameters (105). Increased ammonia or nitrite increased mortality of *H. diversicolor* infected with *V. parahaemolyticus* by reducing immune function (106, 107) and in (23), a difference of only 1°C in temperature had a highly significant impact on mortality level.

Life stage can also be an important factor in pathogenicity of gastropod infectious disease, e.g. immature abalone were insensitive to *V. harveyi*, while ripe or postspawning abalone were susceptible to infection and mortality (23). The abalone reproductive cycle was also an important factor associated with mortalities in (21, 108, 109), also suggested that susceptibility to this pathogen is driven by both climatic factors and reproductive physiology while (42), showed that stress factors had likely precipitated *Vibrio* sp. outbreaks among *H. rubra*, *H. laevigata* and their hybrids.

Life stage (79) and temperature (80) have also been shown to affect the infectivity of *Schistosoma mansoni* to *Biomphalaria glabrata*, which may lead to potential consequences for human health linked to global warming.

There have also been differences noted in mortality between wild and cultured abalone. In (23), the mortality rate due to *V. harveyi* was faster for the farmed than for the wild abalone. The reasons for this difference could include increased stress or reduced genetic diversity in farmed populations (110).

Dang et al. (111) suggested that, in *H. laevigata*, diet may enhance antibacterial activity against *Vibrio anguillarum* wheras (41) found that *Vibrio* sp. was transmitted from the seaweed (*Gracilaria changii*) used as food for the abalone which led to a mortality event in *H. asinina*. This shows that good farming and management practices as well as appropriate husbandry are vital in reducing the spread of pathogenic diseases.

As the number and variety of species of gastropod kept in captivity increases, and the conservation status of further wild populations becomes more critical, research into gastropod diseases, mitigation factors and greater depth of knowledge of those diseases already reported but not fully categorised will take place. This will benefit all gastropods, and hopefully in particular those of conservation importance.

## Author Contributions

The two authors (MO’B and SP) have contributed equally to this work. All authors contributed to the article and approved the submitted version.

## Conflict of Interest

The authors declare that the research was conducted in the absence of any commercial or financial relationships that could be construed as a potential conflict of interest.

## Publisher’s Note

All claims expressed in this article are solely those of the authors and do not necessarily represent those of their affiliated organizations, or those of the publisher, the editors and the reviewers. Any product that may be evaluated in this article, or claim that may be made by its manufacturer, is not guaranteed or endorsed by the publisher.
